# Pit and fissure sealants in dental public health – application criteria and general policy in Finland

**DOI:** 10.1186/1472-6831-9-5

**Published:** 2009-02-04

**Authors:** Sari Kervanto-Seppälä, Ilpo Pietilä, Jukka H Meurman, Eero Kerosuo

**Affiliations:** 1 Institute of Dentistry, University of Helsinki, Helsinki, Finland; 2 Public Dental Health Centre, Pori, Finland; 3 Department of Oral and Maxillofacial Diseases, Helsinki University Central Hospital, Helsinki, Finland; 4 Institute of Dentistry, University of Turku, Finland; 5 Department of Clinical Odontology, Faculty of Medicine, University of Tromsø, Tromsø, Norway

## Abstract

**Background:**

Pit and fissure sealants (sealants) are widely used as a non-operative preventive method in public dental health in Finland. Most children under 19 years of age attend the community-organized dental health services free of charge. The aims of this study were to find out to what extent sealants were applied, what the attitudes of dental professionals towards sealant application were, and whether any existing sealant policies could be detected among the health centres or among the respondents in general. The study evaluated changes that had taken place in the policies used during a ten year period (1991–2001).

**Methods:**

A questionnaire was mailed to each chief dental officer (CDO) of the 265 public dental health centres in Finland, and to a group of general dentists (GDP) applying sealants in these health centres, giving a total of 434 questionnaires with 22 questions. The response rate was 80% (N = 342).

**Results:**

A majority of the respondents reported to application of sealants on a systematic basis for children with increased caries risk. The criteria for applying sealants and the actual strategies seemed to vary locally between the dentists within the health centres and between the health centres nationwide. The majority of respondents believed sealants had short- and long-term effects. The overall use of sealants decreased towards the end of the ten year period. The health centres (N = 28) choosing criteria to seal over detected or suspected enamel caries lesion had a DMFT value of 1.0 (SD ± 0.49) at age 12 (year 2000) compared to a value of 1.2 (SD ± 0.47) for those health centres (N = 177) applying sealants by alternative criteria (t-test, p < 0.05).

**Conclusion:**

There seems to be a need for defined guidelines for sealant application criteria and policy both locally and nationwide. Occlusal caries management may be improved by shifting the sealant policy from the traditional approach of prevention to interception, i.e. applying the sealants over detected or suspected enamel caries lesions instead of sealing sound teeth.

## Background

Dental care in Finland is provided both by private dentists and by community-organized public health centres. The public dental health centres are publicly funded and community-based, providing dental services to all age groups. About half of Finland's 5.2 million inhabitants use private dental services while most children under 19 years of age use the public service. All age groups up to age 19 receive dental services free of charge while other groups pay subsidised fees for treatment. The public health services in Finland are regionally distributed according to the population density of each area. All public dental health centres set their own health care criteria and strategies locally; however, the focus has been strongly on the non-operative preventive care for children. Pit and fissure sealants (sealants) are used by the public health service but neither national guidelines nor general sealant protocols have been published.

Pits and fissures of permanent molars are vulnerable sites for caries lesions due to morphology and plaque accumulation [1,2]. Sealants applied to pits and fissures act as mechanical barriers between enamel surface and the biofilm, and if retained completely, have been shown to be very effective in restricting the growth of bacteria. The studies of Handelman [3,4] from over 30 years ago and some later studies by Mertz-Fairhurst et al. [5,6] have shown that when caries lesions are sealed, the lesion does not progress. Until the middle of 1980's sealants were generally applied in a preventive manner solely to intact, unstained fissures with no suspected enamel caries lesions [7,8]. The present recommendations for sealant application [9-12] relate back to several international consensus reports from the 1980's and 1990's where sealing over enamel lesions and questionable fissures was suggested [13-18].

The selected study period was particularly interesting since after 2001, a new national legislation in Finland changed the focus of public dental health care, extending the system to cover the whole population and thus limiting the resources available for younger age groups. The changed legislation implicated a veritable increase of costs for the publicly funded oral health care.

The aim of this study was to find out to what extent the earlier guidelines and recommendations (published up to 1995) [13-21] were adopted by the dental professionals in the Finnish dental public health system. Attitudes towards sealants, as well as changes in the attitudes were recorded during the studied period from 1991 to 2001. Moreover, we wanted to determine the frequency of sealant use among other preventive or interceptive procedures in dental public health. Furthermore, our purpose was to determine whether uniform criteria and policies for sealing or locally agreed sealant strategies could be found. The specific aim was to find out whether a relationship between past caries experience and the sealant application protocol used could be found within the health centres.

## Methods

A structured questionnaire was mailed to each of the 256 public dental health centres in Finland during the year 2001. The questionnaire covered demographic items, examination policies, sealant application protocol, changes in oral health practice over the studied period, attitudes towards sealant application and sealant efficacy, and the local DMFT index values of each health centre. For the present study, the data were categorized as follows:

- Demographic data: occupation/status (CDO or GDP); location and size of the health centre where each respondent was working

- Examination policy: annual or individual check-up periods

- Sealant application protocol: local agreements on sealant application criteria, sealant application criteria and protocol, sealant materials in use, caries-risk evaluation

- Changes in protocols (examination policies, sealant application)

- Attitudes towards sealant use, sealant efficacy and the plausible costs of sealant application procedures

- Local DMFT index values of each health centre (years 1991 and 2000)

The questionnaire was initially piloted by three CDOs and was amended according to their suggestions before the study began. Public health centres collect data of the patients examined and treated: In Finland the DMFT index value is recorded from all patients at every examination and the DMFT of all patients monitored by age groups.

As the population density varies greatly regionally, we categorized the health centres into subgroups of 'large' and 'small' in order to get a representative cluster sample among the dentists applying sealants at the health centres. Consequently, public dental health centres with less than 7 dentists were classified as 'small' while all other health centres were classified as 'large'. The number of dental hygienists or dental nurses did not affect this classification.

In all cases the questionnaires were mailed to the chief dental officer (CDO). An additional questionnaire was mailed to every 7^th ^general practitioner (GDP) in the 'large' health centre-group. These additional questionnaires were addressed especially to dentists applying sealants; these dentists were identified locally by each CDO. Thus dental surgeons and orthodontists, for example, who do not apply sealants, were excluded from this sample. The questionnaire was simultaneously sent by e-mail so the respondent could choose the most convenient way to reply.

The 'large' dental health centres (N = 77) received a total of 254 questionnaires (each health centre receiving 2–29 questionnaires) while the 180 health centres that fell into the 'small' – category received only one questionnaire each. A sample of 434 questionnaires was issued to the CDOs: 267 (62%) to be replied to by him/herself while 167 (38%) questionnaires were requested to be delivered forward to a GDP in that health centre.

### Sealant application protocol: criteria and policies

In this study the systematic use of sealants was defined as follows: "Sealants are taken into consideration as a possible treatment mode and are usually applied to teeth according to particular criteria." Even though the final decision regarding sealant application was always made by the operator himself, information regarding any existing local agreement on the general guidelines for the criteria was requested from each respondent. Sealant application criteria and the policies used were recorded. Information on the treatment of choice was also requested in some specified situations, for example in the case of partially erupted molars at risk for dentin caries. Factors indicating low caries risk were scored in a question further evaluating the risk of dentin caries development in permanent molars. The type of sealant material used was recorded as well as changes in the material choices between 1991 and 2001. Reasons for totally abstaining from sealant application as well as the use of other preferred non-operative procedures in dentin caries prevention and management were recorded.

### Dental examination periods and the DMFT-values

The check-up intervals, as well as the criteria for choosing either a fixed (annual) or an individual examination interval, were recorded (years 1991 and 2001 respectively). Based on the examinations of the 12-year old children in 1991 and 2000, the DMFT index values were collected from each participating health centre. The change (decrease or increase) in the DMFT index values between 1991 and 2000 of each dental health centre was evaluated as well as the relationship between the systematic sealing and the DMFT-value in 2000. To find out the impact of the interceptive policy (sealing over enamel caries) on the prevalence of dentinal caries, the DMFT rates in year 2000 of those health centres that reported to have applied this policy in 1991 were compared to the year 2000 DMFT rates of the rest of the health centres applying an alternative sealant policy. T-test was used for the statistical analysis.

### Profession of the operator

The respondents were asked to indicate whether the sealants in the health centre were applied by dentists or by dental auxiliaries both in 1991 and 2001. In cases where dental hygienists or dental assistants applied sealants the respondents were asked who was responsible for the final treatment decision, the dentist or the auxiliary.

### Efficacy and of sealants in relation to the costs

The respondents were asked to evaluate the outcome after sealant application (short- or long-term efficacy of sealants) as well as the costs implied from the sealant approach. The value of an intact tooth achieved by an efficient sealant program was estimated by a hypothetical question where the intact tooth was compared to an adequately restored one; the respondent was asked for his willingness to pay for the costs of sealant procedures.

## Results

Of the 434 issued questionnaires, a total of 342 were returned after one re-issue to the non-responders, giving a response rate of 79%. The small health centres returned 85% (N = 153), CDOs of the large health centres 70% (N = 60) and the GDPs of the large health centres 77% (N = 129) of the questionnaires, respectively. For a cluster sample, where health centre makes up a cluster, the response rate was 85% (N = 219). Of all the responses given, CDO's replies comprised 58% (N = 199) and GDP's 42% (N = 143), respectively. Four replies given by dental hygienists were included in the GDP-group.

### Systematic use of sealants

**Figure 1:**
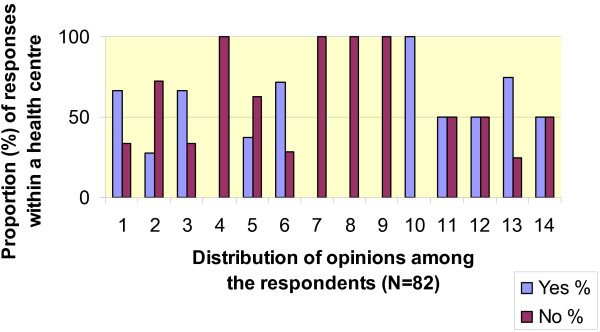
**Systematic sealant use and the distribution rate of opinions within health centres**. Data were collected from the responses from the 14 largest health centres in Finland. The responses (N = 82) from CDOs and GDPs are pooled; the number of questionnaires returned per health centre varied between 2 and 18.

A majority of the CDOs (57%) reported systematic sealant application; among the GDPs this was the case in 48%. A total 44% of dentists working in small health centres, and 64% working in large health centres reported systematic sealant application. On the issue of systematic sealing there was inconsistency in the respondents' opinions within particular health centres. In the large health centres the opinions varied among the CDOs, among the GDPs and between these two groups. Only in five out of the 14 largest health centres did all respondents give a consistent answer to the question of whether sealants were used systematically (Fig. 1). Among the respondents reporting systematic sealant use, 49% had agreed on a local sealant policy including criteria for when to apply sealants. In most cases this agreement was verbal; a written document on the intended sealant policy was only found in 15% of the small health centres and in 5% of the large ones. In majority of health centres the agreement had been taken into practise in 1990 or earlier; 32% of the respondents reported that the criteria had been amended afterwards.

### Sealant application criteria and protocol

The respondents from small health centres applied sealants more extensively on suspected or detected enamel caries in 1991 than did those from large health centres. During the ten-year period, a distinct shift of sealing over on enamel caries lesions had taken place: the proportion of respondents using this criteria increased from 30% in 1991 to 37% in 2001, yet 44% preferred to seal only the sound fissures in 2001 (Table 1).

**Table 1:** Sealant application criteria for molar fissures

**Criteria for sealant application**	**Large**		**Small**		**All**	
	**1991**	**2001**	**1991**	**2001**	**1991**	**2001**
Unstained and Intact Fissures	17	20	18	17	18	18

As above + Stained Fissures	35	21	42	32	38	26

As above + Suspected or Detected Enamel Caries	20	27	29	39	25	33

As above + Suspected or Detected Dentin Caries	5	3	5	6	5	5

No Specific Policy	17	18	6	3	11	11

Alternative or Unknown Policy	7	11	0	3	3	7

Total	**100**	**100**	**100**	**100**	**100**	**100**

Distribution of responses (%) from large (N = 60) and small (N = 66) health centres in 1991 and 2001.

In permanent molars with suspected or detected enamel caries lesions at the occlusal surface, the most common choice of treatment in 2001 was to open the fissure up at the enamel level and to apply a sealant. The preceding eradication of enamel caries before applying a sealant was done almost as often as the application of topical fluoride to suspected occlusal surfaces. The simple sealant application procedure had further lost its popularity in 2001 (Table 2). In those 28 health centres reporting application of sealants on suspected or detected enamel caries lesions in 1991, the DMFT value in 2000 was 1.0 (SD ± 0.49) at age 12 compared to a value of 1.2 (SD ± 0.47) for those health centres (N = 177) applying sealants by alternative criteria (t-test, p < 0.05).

**Table 2:** Proportions of treatments chosen in 1991 and 2001 (%)

**Treatment of choice**	**All respondents**	
	**1991 (N = 96)**	**2001 (N = 91)**

No Procedure	2	2

Re-examination after a Shorter Period	2	0

Fluoride Application on Fissure	13	9

Fluoride Application on Fissure and Re-examination after a Shorter Period	13	24

Sealant Application	11	7

Sealant Application and Re-examination after a Shorter Period	3	3

Sealant Application after Opening the Enamel with a Bur	43	34

Preventive Resin Restoration (PRR) after Opening the Fissure up to Dentin	8	10

Filling	3	7

Alternative or Unknown Policy	2	4

Total	**100**	**100**

The intended treatment of choice for suspected or detected enamel caries lesions on molar occlusal surfaces.

Most of the respondents applied sealants to both the first and second permanent molars in 1991 and 2001. The tendency not to choose selected target teeth for sealant application increased towards the end of the study period (Table 3).

**Table 3:** Distribution of the teeth groups chosen for sealant application in 1991 and 2001(%)

**Teeth groups to which sealants were applied**	**Large**		**Small**		**All**	
	**1991**	**2001**	**1991**	**2001**	**1991**	**2001**
First and Second Permanent Molars	85	69	70	71	77	70

First Permanent Molars	5	7	15	12	10	10

No Teeth Groups Specified	0	11	0	8	0	10

Second Permanent Molars	4	11	3	0	4	5

First and Second Permanent Molars, Premolars	2	0	8	5	5	3

First and Second Permanent Molars, Premolars, Others	4	0	2	2	3	1

Second Permanent Molars, Premolars, Others	0	2	2	0	1	0

Teeth Groups Other than the Above	0	0	0	2	0	1

Total	**100**	**100**	**100**	**100**	**100**	**100**

All respondents applied sealants on a systematic basis. The values are sorted according to the size of the health centre. N = 113.

### Erupting molars in caries risk

A total of 41% of the respondents reported not to have used any specific treatment policy for erupting molars at risk for caries in 1991. By 2001 the proportion of respondents lacking any special policy in such cases had increased to 52%. Of the respondents, 29% in 1991 and 25% in 2001, respectively, applied topical fluoride once to the fissure as a treatment. About one-fifth of the respondents sealed the visible part of the fissure in both 1991 (22%) and 2001 (19%). In such cases the preferred maintenance period was not changed by the majority of respondents. Re-scheduling the following examination to an earlier appointment was chosen by 42% of the respondents both in 1991 and 2001, respectively.

### Examination policies

Most of the respondents (91%) reported examining children annually in 1991 irrespective of their dental status. In 2001 this was the case 17% of the time while 78% reported individually determined intervals for dental examinations. In some health centres a dental hygienist first examined the patient but had the opportunity to consult the dentist before making the decision on whether to seal or not to seal.

If a previously applied sealant was found defective at examination, this did not usually lead to further maintenance. Re-evaluation of sealants and necessary resealing was reported by 26% (1991) and 8% (2001) of the respondents – this was the second choice treatment in 1991 and in 2001. Maintenance and re-maintenance of sealants and the sealed teeth was considered unnecessary by 29% of the respondents in 1991 and 37% in 2001, respectively.

### Evaluation of dentin caries risk

The most obvious predictor reported to indicate low risk for dentin caries at fissures was the intact dentition of the child (52%). The second predictor of choice was a good observed level of dental hygiene (44%) and the third factor reported (41%) was the observation of gently sloping cusps of molar teeth (shallow fissures). Over one-third of the respondents thought that the absence of initial (enamel) caries was a good indicator and over one-fifth that the absence of visible plaque or gingival bleeding would indicate low dentin caries risk. The reported indicators of least importance were gender, lack of visible calculus, lack of use of dental floss and the overall caries decline among children.

### Sealant material

The material of choice was resin-based (RB) light-cured composite in both 1991 and 2001: it was chosen by 68% of the respondents in 1991 and by 83% in 2001. Glass ionomer (GIC) sealants maintained their minor proportion of usage (14% in 1991 and 2001) throughout the 10 year period, whereas the use of chemically-cured RB composite materials had diminished to a negligible level by the year 2001. A small minority of the respondents (1% in 1991; 4% in 2001) reported the use of other material in fissure sealing; these materials were RB flow composites or compomers.

### Reasons for refraining from the use of sealants

Ten percent of the respondents refrained totally from applying sealants. Those dentists or health centres that did not apply sealants at all gave several reasons for this. The main reason was that sealants were thought to have low cost-effectiveness (30%). One-fourth of the respondents (26%) thought that there was no further need to seal fissures since the local DMFT values had decreased to the low levels they were then. Nearly one-fifth (17%) shared the opinion that sealants were ineffective or that other methods were more effective than sealants in arresting enamel caries lesions at occlusal surfaces.

### Application of sealants by dental auxiliaries

**Figure 2:**
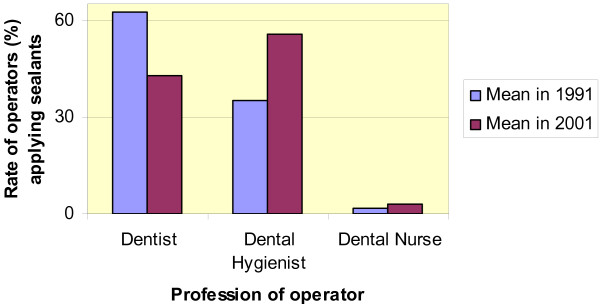
**Distribution of applied sealants by profession**. Means of respondents' estimates for 1991 (N = 306) and 2001 (N = 327).

The estimated number of appointments with dental hygienists increased both with respect to independent decision-making and to the actual procedure of sealant application. In 1991 the majority of sealants were applied by dentists, but by 2001 the dentists were outnumbered by dental hygienists (Fig. 2). In health centres where sealants were applied by dental auxiliaries, the dentist examined and set the initial diagnosis in 69% of the cases in 1991 and in 47% in 2001, respectively. A small minority of health centres reported dental assistants as the main group applying sealants.

### Role of sealants in caries prevention and management

Most of the respondents estimated that sealants had both long- and short-term effects on dentin caries development (Table 4). When asked the hypothetical question of what should be done if the treatment would concern their own child, one-third of the respondents (N = 98) were willing to pay whatever was needed to cover the costs to ensure intact teeth rather than receiving a filling free of charge.

**Table 4:** Short- and long-term effectiveness of sealants

**Definitions best describing the short- and long-term effectiveness of sealants**	**%**	**N**	**Score**
In **some **cases sealants can prevent the development of dentin caries for **a lifetime**	70	209	1

In **most **cases sealants can prevent the development of dentin caries for **some **years	42	145	2

In **most **cases sealants can prevent the development of dentin caries for **a lifetime**	21	71	3

In **some **cases sealants can prevent the development of dentin caries for **some **years	18	63	4

Sealants have **more short**-than long-**term effects **on caries development on the occlusal surfaces	16	56	5

Sealants have **more long**-than short-**term effects **on caries development on the occlusal surfaces	12	40	6

Other opinion	3	10	7

Sealants **do not **have **long-term effects **on caries development on the occlusal surfaces	2	6	8

Sealants **do not **have **short-term effects **on caries development on the occlusal surfaces/No opinion	1	2	9

Pooled distribution of opinions. As multiple choices were possible, the sum of given responses (N = 602) exceeds the number of respondents (N = 343).

Of all the appointments for children up to age 19, the estimated proportion of appointments where sealants were applied decreased from 16% in 1991 to 10% in 2001. A vast majority (76%) of the respondents estimated that they had applied fewer sealants towards the end of the 10-year period of 1991–2001. Most respondents (96% in 1991; 97% in 2001) reported using other methods in addition to sealant application for the prevention and management of pit and fissure caries. The prevailing method of choice was fluoride therapy, either in topical form or as fluoride tablets.

## Discussion

Even though sealants have been widely used in Finland since 1970, neither uniform criteria for sealant application nor a trend regarding sealant policy could be found among the responses in this present study. Only a few health centres had defined the criteria and a policy for sealant application by local agreement.

This study gives information about the gradual changes that have taken place in dental public health in Finland during the 10-year period. A questionnaire study has its limits since most of the responses are self-reported and do not give exact information. As some of the questions go back to the situation over ten years ago, the information should be interpreted with caution. However, it can be assumed that the trends of the attempted sealant approach will stay in mind even if the details are forgotten, thus this study demonstrates attitudes towards sealant application and describes the sealant policies in practice.

With susceptible fissures, sealing on enamel caries was the expected treatment of choice since it has been shown to efficiently restrict the growth of bacteria in the occlusal lesion [1,3-6,22]. Although one-third of the respondents included suspected or detected enamel caries in the application criteria for sealants (Table 1), only a minority of them intended to place the sealant in an interceptive manner. On the contrary, a vast majority of those respondents reporting sealing as an option for management of suspected or detected enamel caries (Table 2) would have applied a sealant only after first cleaning and widening the fissure, and thus eliminating the initial lesion with a rotary instrument. The procedure resembles application of preventive resin restoration (PRR) as was first suggested by Simonsen [23]: the susceptible fissures at occlusal surfaces are opened up with a small tapered fissure bur prior to restoring the cavity with diluted composite. RB sealant is then applied over the edges of the filled cavity, covering also the other remaining pits and fissures at the occlusal surface.

In terms of resource requirements, this treatment modality (PRR) is almost as time-consuming and personnel-demanding as a sealant restoration extending to the dentin [24], and is thus not comparable with interceptive sealant application. Moreover, opening the susceptible fissure is no longer considered necessary, since sealants have been shown to be effective when placed in a cariostatic manner, thus arresting the progression of the eventual enamel lesion [3-5,11,18]. Early caries management by sealant application is recommended in recent consensus statements [9,12]. Only cases where the caries lesion has with certainty progressed to dentin is restorative treatment advocated, preferably in the form of sealant restoration [16,18].

During the ten-year period a definite shift from annual examinations towards fixed individual examination intervals was found. The number of dental auxiliaries, mainly dental hygienists, participating in both independent decision-making and the actual procedure of sealant application increased. We believe that this is a continuing trend which allocates more of the preventive and interceptive procedures to dental hygienists.

A majority of the respondents applied sealants to both the first and second permanent molars. This is in line with the earlier studies of Bohannan et al. [1], who showed that the permanent first and second molars are several times more susceptible to decay than the premolars. Most respondents did not have any treatment policy for erupting molars at risk for caries even though the erupting molars are vulnerable to develop dentin caries due to plaque accumulation, as was reported by Carvalho et al. [2] with erupting first molars.

Re-examination at six-month intervals was shown in the studies of Vehkalahti et al. [25] to be most beneficial to the erupting first permanent molars since the fissures at risk could be sealed soon after eruption. Even a self-diagnosed high dentin caries risk of the erupting molars did not change the intended maintenance period for most of the respondents in this study. With erupting molars at risk for caries, the respondents frequently applied topical fluoride to the occlusal surfaces even though fluoride varnish applied topically to the fissure did not markedly reduce the rate of caries in the studies of Holm et al. [26]. Bravo et al. [27] compared fissure sealing and fluoride varnish application on first permanent molars and found sealant application more effective in dentin caries prevention even though the fissures were sound prior to sealing. This is also in line with the meta-analysis of Hiiri et al. [28], who found pit and fissure sealants superior to topical fluoride.

Increased caries risk led a majority of the respondents to consider sealant application to molar teeth, which is in line with the conclusions of Beauchamp et al. [12], who found that sealant application to high risk individuals was effective. They also recommended periodic reconsideration of caries risk status. Kumar et al. [29] targeted sealant application to high-risk first molars on a school-based program and found this approach effective when compared to unsealed low-risk first molars. Targeting preventive procedures according to individual risk-assessment has been criticized as being impractical and thus inefficient in dental public health by Burt [30]; he concluded that, as the risk assessment methods are imprecise, persons at risk cannot be adequately identified. Unacceptable precision in caries prediction in general was also found in the studies of Alanen et al., where the dental clinicians tried to identify the high-risk subjects. They concluded, though, that some experienced clinicians were able to predict caries risk with high specifity and sensitivity levels [31]. Nevertheless, shifting the sealant policy towards a more interceptive procedure in arresting clearly observed changes (suspected or detected enamel caries lesion in the fissure) would at least partly overcome this dilemma.

GIC remained the material of choice for 14% of the respondents in 2001, even though several studies have found the retention rate of GIC sealants lower than that of RB sealants [22,32-34]. It is concluded that sealants are very effective in preventing dentin caries if completely retained on the tooth surface. To remain the sealant integrity, recall and maintenance of sealants and sealed teeth is necessary [9,10,12]. Re-examinations and resealing are also suggested in the studies of Whyte et al. [35] who had clinical success rates of 97–99% with low resealing rate in their sealant study. They found that dentin caries formation occurred every year and re-examinations and resealing was suggested for children at risk for dentin caries.

There was an overall decrease in the DMFT values of the 12-year old children from 1.5 to 1.2 during the period studied. Low DMFT-index values were found more often in the health centres where sealants were applied over suspected or detected enamel caries. Therefore, caries management of incipient enamel lesions at the occlusal surfaces seems to be more effective than caries prevention at these sites. One reason why dentists are afraid of applying sealants is probably the fact that they are not willing to leave carious tissue under a sealant or they lack the knowledge of arresting incipient caries lesions by simple sealant application. With defined criteria and a protocol for sealant application, the dentists in public health centres can probably be encouraged to use sealants more often and to concentrate on the arresting of lesions. The results of the present study may be modified by the fact that in year 2000 the children were no longer examined at the age of 12 as a total age-group. As caries shows a skewed distribution, the children considered to be in the high-risk group are probably examined and treated more often than others not at risk. Even though the DMFT values (in years 1991 and 2000) are thus not comparable with each other, they do reflect the general trend and the changes found in each health centre.

The present study showed vast variation in the adopted sealant policies; the DMFT data was in line with earlier recommendations favouring the interceptive approach. Rather than caries prevention of sound teeth, the use of sealants should be restricted to the non-cavitated enamel lesion in order to arrest the growth of the bacteria and thus to prevent the initial lesions from progressing into dentin caries. As substantial amount of resources, time and effort are required for the preventive/interceptive dental procedures, it is important to use those resources as effectively as possible.

## Conclusion

The present study suggests that the appropriate sealant policy may have an impact on the dentin caries decline. The use of sealants declined during the studied period; however, majority of the respondents still applied sealants in 2001 but not in an interceptive manner even though this was suggested by the evidence based recommendations since 1980's.

## Abbreviations

CDO: chief dental officer; DMFT: decayed, missing or filled permanent teeth; GIC: glass ionomer cement; GDP: general dental practitioner; PRR: preventive resin restoration; RB: resin-based material.

## Competing interests

The authors declare that they have no competing interests.

## Authors' contributions

SK participated in the study design, carried out the study, analyzed the data and wrote the manuscript. IP participated in the design of the study and tested the questionnaire at a public health centre before starting the study. JHM interpreted the data and contributed to drafting the manuscript. EK conceived of the study, participated in the design and analysis of the study, performed and interpreted the statistical analysis, and helped to draft the manuscript. All authors read and approved the final manuscript.

## Pre-publication history

The pre-publication history for this paper can be accessed here:


